# An exploratory study on rabies exposure through contact tracing in a rural area near Bengaluru, Karnataka, India

**DOI:** 10.1371/journal.pntd.0006682

**Published:** 2018-08-06

**Authors:** N. R. Ramesh Masthi, Pruthvi S.

**Affiliations:** 1 Department of Community Medicine, Kempegowda Institute of Medical Sciences, Bengaluru, Karnataka, India; 2 Department of Community Medicine, Sapthagiri Institute of Medical Sciences, Bengaluru, Karnataka, India; Wistar Institute, UNITED STATES

## Abstract

**Need for study:**

Rabies is a neglected zoonotic disease. Given the low incidence, apart from the existing reporting syst, there is a need to look for other means of case detection strategies for rabies. Contact tracing is one such method to efficiently capture information.

**Objectives:**

To find out the rabid status of biting animal through contact tracing and to determine health seeking behavior of the bite victims.

**Materials and methods:**

An exploratory study using contact tracing was conducted during the first quarter of 2017 in villages coming under three Public Health Centers. The households of the bite victims were visited and details of rabies exposure obtained from the bite victim/ adult responsible respondent using a standardized questionnaire.

**Results:**

A total of 69 dog/cat bite cases were identified. 69.5% of bites were by stray dogs. 97.1% bite victims had Category III bites. Only 4.5% bite victims had taken PEP. 70.1% of animal bite cases were administered ARV. Only 7.2% bite victims had exposure to probable rabid animals. All dog bite victims were alive after 3 months of follow up.

**Conclusion:**

Contact tracing was successful in case detection of probable rabid animal exposures and suitable for a period of one year.

## Author summary

In India, Rabies is a neglected zoonotic disease and the burden of rabies is usually not captured by the health system due to varied reasons. Hence, an exploratory study was attempted to find out the rabid status of the biting animal through contact tracing during the first quarter of 2017 in villages coming under three Public Health Centers. A total of 69 dog/cat bite cases were identified. Majority of bites were by stray dogs, Category-III, exposure to non cases and few received PEP. Percentage of bite victims who actually required PEP was calculated. These findings support the fact that contact tracing can be used as an additional tool by the health care provider for measurement of rabid animal bites, where resources are scarce, reporting systems are weak and priorities for disease vary. A rabies check list/score card to be made available at Public Health Centers, which shall ensure better utilization of limited but lifesaving Rabies Immunobiologicals.

## Introduction

Rabies is a 100% fatal *viral zoonotic* disease.[1] However, *Rabies* can be prevented if, health care providers are able to identify the type of exposure, categorize the wounds and provide post exposure prophylaxis(PEP) as early as possible.[2,3] South East Asia region accounts approximately for 60% of human rabies deaths in the world. An estimated 20,000 rabies deaths (*approximately 2/100*,*000 population*) and 17.4 million exposures to animal bite occur every year in India.[4]

The data on incidence of human and canine rabies is currently not known and needs to be updated. In reality, the burden of rabies is usually not captured by the health system due to varied reasons. Moreover, *Rabies* is not a notifiable disease in India and acts as a surveillance barrier for measuring the burden of disease. Also, the laboratory-based animal rabies surveillance program to measure the burden is non-existent in many regions of the country.

Human and animal rabies cases are under reported in most of the developing countries due to ineffective rabies surveillance methods.[5] To understand the national burden of rabies, estimation methods must be periodically conducted.[6]In India, the populations most at risk from rabies exposures are living in rural areas, in poor, marginalized communities where surveillance and reporting systems are weak. The need of the hour is measurement of bite victims bitten by a rabid dog and having access to PEP. Contact tracing provides an efficient method of documenting such information. Contact tracing consists of sequential steps of data collection and investigation, revealing different aspects of health seeking behavior, treatment cost and health outcomes. Contact tracing is finding everyone who comes in direct contact with rabies exposure usually occurring through dogs and helps in targeting at risk population [7]

In India, A number of changes have taken place over the years including introduction of *intra dermal rabies vaccine* (IDRV), abolition of nerve tissue vaccine, availability and accessibility to *rabies immunobiologicals* and better awareness about rabies in the population. [8]Hence it is assumed that the rabies burden would have come down.

In this background, an attempt has been made to find out the rabid status of the biting animal through contact tracing in a rural area, to determine the health seeking behavior of the bite victims and to recommend measures for rabies prevention.

## Methods

**Fig 1:**
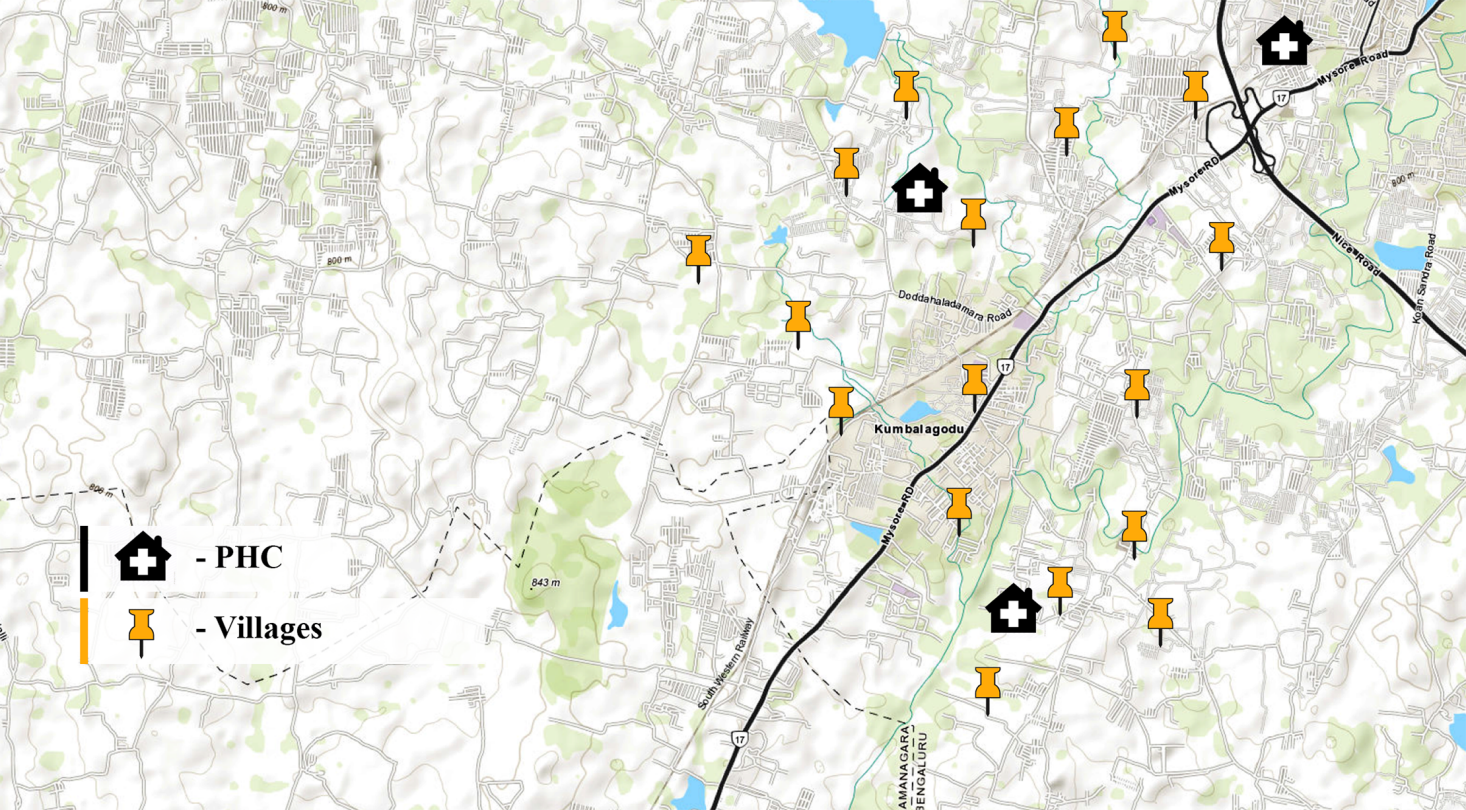
Spatial map of the study area. **(**USGS landsatlook [online].Available from https://landsatlook.usgs.gov/viewer.html.[Accessed on 13.06.2018]).

An exploratory study was conducted during the first quarter of 2017 in villages (rural area) coming under three Public Health Centers (K.Golahally, Kengeri and Sullikere). The villages were located about 25 kilometers away from Bengaluru, Karnataka, India. The average population was about 1100 and population of the villages ranged from 81 to 3165. The study area is located in a dry belt with little agriculture activity and the mean temperature was about 25°C. [9] Map of study area is described in Fig 1.

Typical contact tracing includes diseases which involves human to human transmission. As far as rabies is concerned, one of the participants in transmission could not be interviewed i.e. the dog. Hence, the methodology for contact tracing was modified, where the index case was interviewed to identify other exposures of the biting animal, which would herewith be considered as contacts.

### Initial contact

The local government primary care provider (Anganwadi Centre) in each village was the starting point of contact tracing. The female staff was the first informant, as she is the front line primary care worker and would have had basic information on the morbidities reported. Through her cooperation, the initial process of identifying the index bite victims was started, which would have otherwise been difficult for the investigator in terms of acceptability and feasibility. The bite victims were people who had been exposed to animal bites in the last one year from the date of survey. Ideally, the bite victims details should have been got from the health center. However, the address and village would have been difficult to get as they are usually not recorded in the health center. Hence, the index case was the starting point of contact tracing in the village. Majority of the bite victims were interviewed within 6 months of the exposure, the minimum and maximum duration of exposure was one week and 11 months from the date of survey. The bite victims were followed up for a period of three months to know the outcome i.e alive/dead.

### Subsequent contact

The household of each bite victim was visited by the investigators and detailed information regarding biting animal, availability of animal, rabies exposure, PEP, rabies-related deaths following animal bites and clinical signs of rabies in the biting animal, etc was obtained using a standardized semi-structured questionnaire. The bite victims were the respondents in majority of the interviews and when they were not available, the head of family /adult responsible respondent were interviewed. For contact tracing of animal bite, the respondents were asked about the index biting animal having bitten other animals or humans and if they had come across people who had been bitten by a dog/cat.[10]Subsequently, the households of other bite victims were visited and detailed information on exposure was obtained. This procedure of contact tracing (snowball sampling technique) was repeated until all probable exposures were identified. To maximize the efforts to trace all bite victims, information was also obtained from villagers, Accredited Social Health Activist (ASHA) workers, formal and informal leaders. Additionally, the investigators searched for the availability of the biting dogs/cat during the survey. The bite victim must have been a resident of the village for a minimum of six months. Subjects bitten by animals not known to cause rabies in humans were excluded from the study. Finally, a total of 69 dog/cat bite victims were selected through contact tracing during first quarter of 2017 covering 17 villages.

### Surveillance case definitions

#### Confirmed rabies cases: Diagnostic confirmation of rabies virus by DFA

*Probable rabies cases*. Animals that were not tested for rabies and died during observation OR Did not pass observation (escaped animals, not found by further investigation) OR Developed one or more clinical sign and died after being bitten by a suspect/probable/confirmed rabid animal. *Suspect rabies cases*. Animals reported to Animal Rabies Surveillance Officers that could not be assessed OR Animals that had less than 2 signs of rabies and test results were inconclusive. *Non-cases*. Animals that are healthy after the 14-day observation period OR Animals that test negative by DFA.[11]

#### Ethics statement

The ethical clearance was obtained from the ethical committee of Kempegowda Institute of Medical Sciences, Bengaluru. Permission was given for oral consent as study involved only collection of information and did not have interventions. Confidentiality of study participants and data was ensured.

#### Data collection and analysis

Data were entered into a Microsoft excelsheet and exported to Stata 12.1(statistical software). The data was analyzed and descriptive statistics expressed in terms of simple percentages and proportions.

## Results

**Fig 2:**
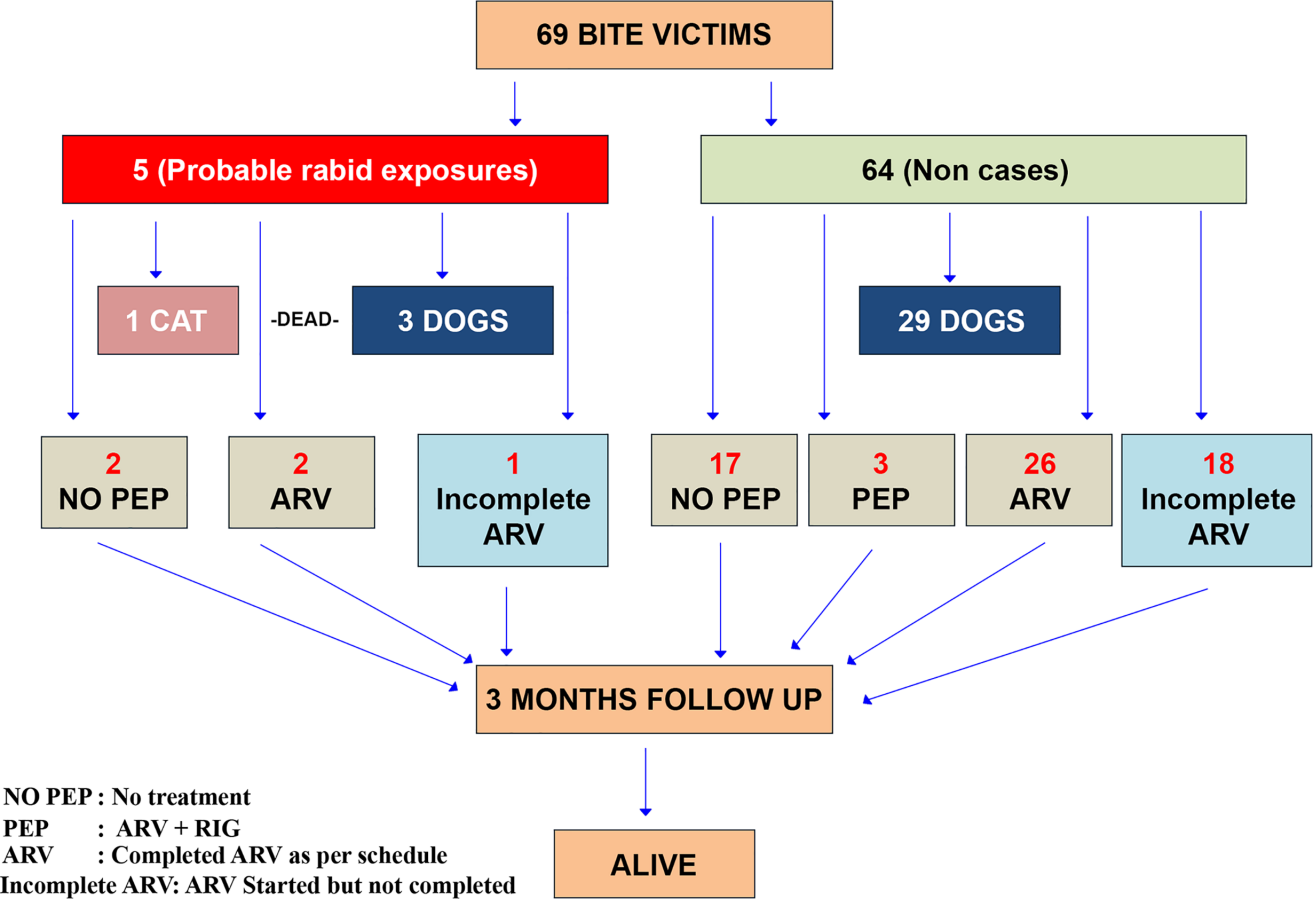
Flow chat of bite victims using contact tracing.

A total of 69 bite victims were followed up. The age range of bite victims was 3 to 89 years respectively. The median age of bite victim with Inter Quartile Range (IQR) was 17 (9,18) years. 52% of the bite victims were males. 33 animals (32 dogs & 1 cat) were responsible for the exposure among 69 bite victims. 69.5% of the bites were by stray dogs. There was one (1.4%) event of a dog having bitten a cat. There were no wild animal bites. Information regarding outcome (alive, dead, sick, unknown) of all the 33 dogs/cat was available and given by the bite victims. Out of the 69, 5(7.2%) bite victims had exposure to 3 probable rabid dogs and 1 probable rabid cat. The remaining bite victims were exposed to non cases. All 4(100.0%) probable rabid dogs/cat had died within 10 days. The clinical signs observed in the probable rabid dogs/cat were hypersalivation in 3(75.0%) and aggressive behavior in 3(75.0%). Laboratory confirmation of rabies diagnosis was not done in any of the dogs/cat that had died. All the 69(100%) bite victims were alive at the end of three months of follow up (Fig 2).

**Fig 3:**
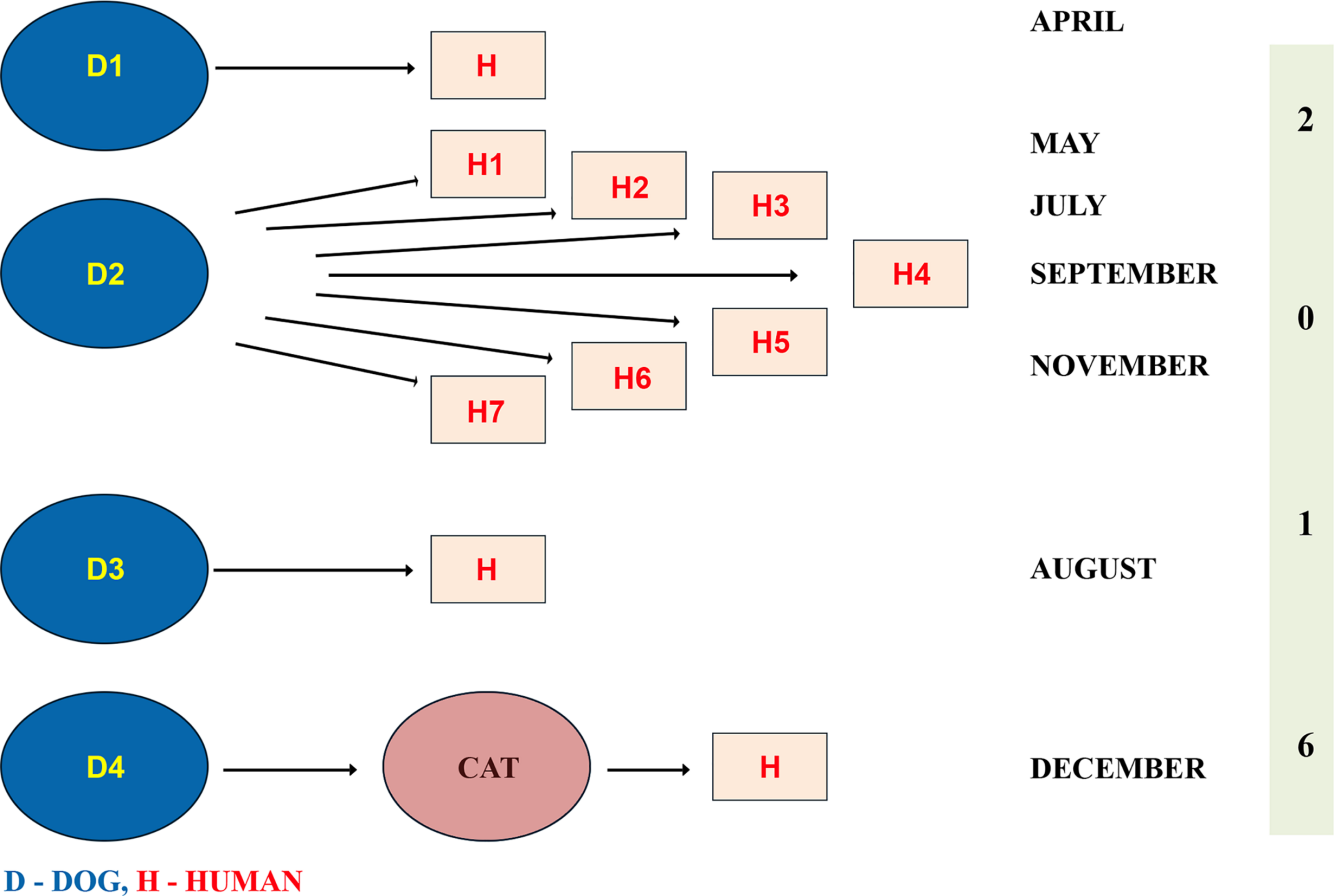
Contact tracing in village-X.

There was no relationship between any two dog bites within the village and between adjacent villages. Each of the bites had occurred in different time periods as sporadic event. In majority of the villages, one dog was responsible for each exposure. A maximum of 7 persons were bitten by a single dog. Only in one village, 4 dogs were responsible for 4 different exposures. An example of contact tracing in village X is given in Fig 3.

67(97.1%) bite victims had category-III exposures and 2(2.9%) had category-II exposures. 3 (4.4%) category-III exposures had received PEP (Rabies Immunogobulin & Anti Rabies Vaccination), 46(68.7%) had received Anti rabies Vaccination (ARV) alone and 18(26.9%) had not sought any treatment. 1(50.0%) category-II bite victims had received ARV and 1 (50.0%) had not sought any treatment. Among those who had received ARV (PEP included), 31(62%) bite victims had completed full course of ARV (5 doses) and 19 (38%) had incomplete Anti Rabies Vaccination.14(73.6%) bite victims informed that doctor /health worker did not advice and 5(26.4%) said busy as reason for not completing ARV. Among the 5 bite victims exposed to probable rabid dogs/cat, the age of the victims were 14,16,17,32,and 60 years. 3 (60.0%) were males and 2(40.0%) were females, all the exposures were unprovoked in nature, 2 (40.0%)bites were over the hand, 1(20.0%) on the back, 1(20.0%) forearm and 1(20.0%) was on the leg. 2 (40%) bite victims had washed the wound with soap and water,1 (20%) washed only with water,1(20%) had applied antiseptic and 1(20%) did not do anything. Two (40%) probable rabid bite victims had completed ARV (5 doses), 1 (20%) had incomplete Anti Rabies Vaccination, while the other 2(20%) had not sought PEP and reason being ignorant of ARV. An average of one dose of ARV was taken by non cases and average of three doses of ARV by probable rabid animal bite victims. The average number of people bitten by probable rabid dogs was one and average number of people bitten by non rabid dog was two. None of the bite victims had received first aid by the village primary care provider and all of them were referred to higher centers. 26(52%) bite victims had visited the private health care provider for availing treatment and 10(20%) had visited both government and private health care centers. The average cost of PEP incurred per person was Rs.1049.70(16$) and average cost of transport Rs.165.24(3$). However, assessment of costs is additional information gathered and was not looked as barrier for PEP. Table 1 describe the ratio of rabies exposure and PEP.

**Table 1:** Ratio of bites and PEP.

Variables	Numbers
Total animal bites	69
Number of non case dogs : Number of bite victims	29: 64
Number of probable rabid dogs : Number of bite victims	3:4
Pet dog bites : Stray dog bites	10:22
Government health care provider : Private health care provider	25:25
ARS with ARV : ARV only	3:48
Number of bite victims alive(End of 3 months) : Dead	69 : 0

## Discussion

### Contact tracing

Contact tracing conducted immediately with little or no delay between the bite and the interview, will give detailed and accurate information. Contact tracing can also be carried out retrospectively to maximum of one year for more reliable data. Data collected beyond one year can result in recall error. [10] From contact tracing it was observed that, less than 10% of the exposures were by probable rabid animals and no brain sample was examined for confirmation of rabies. The interview of index case, family members and stake holders to trace bite victims and animal cases in the villages was similar to the studies done in Bali and Bohol.[12,13] Different studies on contact tracing among community members and healthcare workers, were either to trace bite events, or to look for clinical signs of rabies in the biting dogs, or to see if they could find out strategies for rabies control or to identify contacts for post-exposure prophylaxis to prevent the disease.[14,15,16]

### Bite details and treatment

Majority of the bites were category-III exposures. Thirteen stray dogs were responsible for more than half of the bite victims in concordance to the observations from other studies.[17,18] Majority of bite victims had first visited a clinic/hospital in the town for treatment as the primary care provider in the village was not aware about rabies PEP.

Two (40.0%) probable rabies exposure victims did not seek treatment in the present study compared to contact tracing survey in Tanzania, which had showed that 15% and 24% of suspect rabies exposure did not seek medical attention.[6] In a hospital based study in Bhutan, 32% of the subjects mentioned that the biting dog looked normal and 9% mentioned that the biting dogs looked like suspect rabies contrary to the observation of the present study.[19] Studies have shown that hypersalivation and aggression are the common clinical signs observed for diagnosis of rabies in dogs similar to the finding in the present study. [12,14]

The World Health Organization reported that, almost all rabies death victims had not sought rabies PEP and there were no facilities or health personnel available to provide PEP in many areas where the disease is prevalent and suggested strengthening availability of Rabies Immunobiologicals in these places.[20,7] It is concerning that 60% victims of probable rabid animal exposures were either not aware or, did not seek care or did not complete PEP. The study is too small to make any determinations about the rate or risk of human rabies in the study area.

### Surveillance system

The standard surveillance practices applied to many human and animal diseases consist of case identification, contact tracing, epidemiologic investigation, and laboratory confirmation.[21] The outcome of animal (alive, dead, sick, unknown) was mainly based on information provided by the bite victim/people in the village. The surveillance system for dog rabies diagnosis was non existent in the villages surveyed. The methods of rabies surveillance practiced in many countries suffer from fundamental problems including a lack of trained professionals and lack of diagnostic laboratory capacity. This results in a lack of awareness of case burden, reduced funding for control, and poor community engagement around prevention.[8,11] Having a functioning surveillance system in villages will go a long way in achieving the WHO, OIE and FAO goal to educate, vaccinate and eliminate dog-mediated human rabies deaths in the world by 2030.[22]Laboratory diagnosis is critical to confirm the status of a suspect case, in part, to justify prophylaxis in exposed persons or animals.[23] Human rabies is underreported and the disease is not a priority in endemic countries.[24] One Health emphasizes that the rabies control and elimination should be a joint effort of veterinary and medical field.[25]

The Indian rabies survey had estimated that, for every 870 bites, there will be one rabies case. However, it was not possible to elicit, which bite was responsible for the rabies death.[4] The present study through contact tracing was able to identify bite victims who were exposed to probable rabid animal exposures. India is a vast country with limited resources. Data on availability, accessibility and affordability of PEP is needed to plan for better intervention strategies. Nearly 28% of the subjects did not receive any rabies prophylaxis in the present study. In such a situation, a rabies risk score card based on the knowledge of local rabies transmission, category of bite and dog rabid status, etc can be developed. The rabies score card/check list would be able to identify the bite victims who need rabies PEP. This can be made available to the primary care providers in the village along with campaigns for strengthening of rabies IEC in the community. Yes, there are chances of missing out genuine cases, however these can be overcome if the scale/check list has very high sensitivity, treating physicians are asked to administer PEP in case of doubt and individuals with high risk are targeted.

### Limitations

Snowball methods are typically performed to find rare events, such as human rabies deaths. They are less accurate at describing rates of more common events, like bites or healthcare seeking behaviors. Some of the bite victims may have been missed because of the contact tracing methodology followed (selection bias). Information on details of animal bites, PEP seeking behavior and follow up vaccination of the bite victims was based on the history revealed by the bite victim (Information bias). Categorization of bites was according to bite victims observation (observer bias), money spent and treatment taken are as revealed by bite victims (recall bias). The classification of biting animal as non cases and probable rabid is based on facts given by the bite victims/people (information bias). Clinical and laboratory confirmation of rabies in the biting animal was not possible. A study covering a wider geographical area was not possible due to feasibility issues.

### Conclusion

Contact tracing was successful in case detection of probable rabid animal exposures and suitable for a period of one year. In addition, contact tracing identified that only one tenth of the bite victims required PEP.

### Recommendations

Implementation of contact tracing technique for identification of rabid status of the biting animal. To conduct a prospective study in a larger geographic area. Development of a rabies risk score card for the health care provider.

## Supporting information

S1 Checklist STROBE checklist. (DOC)
